# Throat Colonization and Antibiotic Susceptibility of Group a β-Hemolytic Streptococci Among Rheumatic Heart Disease Patients Attending a Cardiac Referral Hospital in Tanzania, a Descriptive Cross-Sectional Study

**DOI:** 10.3389/fsurg.2020.00057

**Published:** 2020-09-17

**Authors:** Sarah Wangilisasi, Pilly Chillo, Delilah Kimambo, Mohammed Janabi, Appolinary Kamuhabwa

**Affiliations:** ^1^Department of Clinical Pharmacy and Pharmacology, School of Pharmacy, Muhimbili University of Health and Allied Sciences, Dar es Salaam, Tanzania; ^2^Department of Internal Medicine (Section of Cardiology), School of Medicine, Muhimbili University of Health and Allied Sciences, Dar es Salaam, Tanzania; ^3^Department of Cardiology, Jakaya Kikwete Cardiac Institute, Dar es Salaam, Tanzania

**Keywords:** rheumatic heart disease, acute rheumatic fever, throat colonization, secondary prophylaxis, Tanzania

## Abstract

**Background:** Secondary prophylaxis against repeated attacks of acute rheumatic fever is an important intervention in patients with rheumatic heart disease (RHD), and it aims to prevent throat infection by group A β-hemolytic streptococcus (GAS); however, its implementation faces many challenges. This study aimed to assess throat colonization, antibiotic susceptibility, and factors associated with GAS colonization among patients with RHD attending care at Jakaya Kikwete Cardiac Institute in Dar-es-Salaam, Tanzania.

**Methods:** A descriptive cross-sectional study of RHD patients attending the Jakaya Kikwete Cardiac Institute was conducted from March to May 2018, where we consecutively enrolled all patients known to have RHD and coming for their regular clinic follow-up. A structured questionnaire was used to obtain patients' sociodemographic information, factors associated with GAS colonization, and status of secondary prophylaxis use and adherence. Throat swabs were taken and cultured to determine the presence of GAS, and isolates of GAS were tested for antibiotic susceptibility using Kirby–Bauer disk diffusion method according to the Clinical and Laboratory Standards Institute version 2015. Antibiotics of interest were chosen according to the Tanzanian Treatment Guidelines.

**Results:** In total, 194 patients with RHD were enrolled, their mean age was 28.4 ± 16.5 years, and 58.2% were females. Only 58 (29.9%) patients were on regular prophylaxis, 39 (20.1%) had stopped taking prophylaxis, whereas 97 (50.0%) had never been on prophylaxis. Throat cultures were positive for GAS in 25 (12.9%) patients. Patients who stopped prophylaxis were 3.26 times more likely to be colonized by GAS when compared to patients on regular prophylaxis. Majority (96%) of GAS isolates were susceptible to penicillin, ceftriaxone, and ciprofloxacin, whereas the highest resistance (20%) was observed with vancomycin. No GAS resistance was observed against penicillin.

**Conclusion:** The prevalence of GAS throat colonization is high among this population and is associated with stopping prophylaxis. The proportion of patients on regular secondary prophylaxis is unacceptably low, and interventions should target both patients' and physicians' barriers to effective secondary prophylaxis.

## Introduction

Rheumatic heart disease (RHD), a complication of acute rheumatic fever (ARF) caused by group A β-hemolytic streptococci (GAS) is a major cause of cardiovascular morbidity and mortality in young people in developing countries (1). Worldwide estimates by the Global Burden of Disease study suggest that at least the disease affects 33 million people globally and causes about 320,000 deaths annually (2). The World Health Organization (WHO) recommends that all patients with confirmed RHD receive secondary prophylaxis against repeated attacks of ARF in order to prevent further valvular damage (3). The purpose is to prevent colonization or infection of the upper respiratory tract with GAS and the development of recurrent attacks of ARF (3). Implementation of effective secondary prophylaxis, however, has proven to be a challenging task because of a number of reasons including limited awareness, inadequate health literacy, missed opportunities for treatment, and poor access to health care, as well as inadequate health-seeking behavior among patients and their parents/guardians (4). There is also limited awareness among medical personnel on the initiation and continuity of prophylaxis (5). This causes low compliance and adherence, resulting in inadequate prophylaxis and failure to eradicate GAS from the throat (6, 7). Furthermore, GAS throat colonization and carriage rate among asymptomatic children has been reported to be high, ranging from 9.7 to 16% in recent studies from sub-Saharan African countries (8–10), indicating a high burden in the communities.

The recommended antibiotic for secondary prophylaxis is intramuscular benzathine penicillin G (BPG) every 3–4 weeks (3). BPG is an effective agent in secondary prevention because of its long half-life, which provides prolonged bactericidal protection from GAS infection (11). For patients allergic to penicillin, the WHO recommends the use of oral sulfadiazine or sulfisoxazole, whereas erythromycin is recommended for patients allergic to both penicillin and sulfa-containing drugs (3). With effective secondary prophylaxis, recurrence and the progression of rheumatic fever to RHD have been shown to be significantly reduced (12, 13). So far, GAS susceptibility to penicillin has been excellent, with studies from both sub-Saharan Africa (7, 8, 10, 14), as well as outside the region (15), reporting GAS to be almost 100% susceptible to penicillin.

In Tanzania, as with other sub-Saharan African countries, RHD is still a major cause of morbidity and mortality especially among children and young adults (16, 17). Generally, mortality from RHD has been reported to be high and is attributed to disease progression, as well as complications arising from RHD (18). However, contrary to WHO recommendations, initiation and adherence to secondary prophylaxis have been reported to be low especially from sub-Saharan African countries (4, 18–20), hence calling for measures to step up efforts to improve adherence to secondary prophylaxis (21). In Tanzania, information on level of adherence to secondary prophylaxis, as well as its outcome (i.e., the rate of GAS throat colonization among RHD patients), is lacking. Furthermore, the antibiotic susceptibility of GAS isolated from RHD patients in our local setting is not known. We therefore aimed to assess throat colonization, antibiotic susceptibility, and factors associated with GAS colonization among RHD patients attending the Jakaya Kikwete Cardiac Institute, which is the highest referral hospital for cardiac diseases in Tanzania.

## Materials and Methods

### Study Area and Period

The study was conducted at the outpatients' clinics of the Jakaya Kikwete cardiac referral hospital, located in Dar es Salaam city in Tanzania. The hospital is the main referral hospital for heart diseases in Tanzania, including patients for open heart surgery, and it is also a teaching hospital for the Muhimbili University of Health and Allied Sciences. The study was conducted between March and May 2018.

### Study Design and Sampling Technique

A descriptive cross-sectional study was conducted. The study included all patients known to have RHD who were coming for their regular clinic follow-up. Patients were consecutively enrolled in the study as they attended the clinics until the sample size was reached. Using previous prevalence (7), a sample size of 194 RHD patient was enough to determine the prevalence of throat colonization at 95% confidence and at an error margin of 5%.

### Clinical Data Collection

A structured questionnaire administered by a trained nurse was used to collect information on patients' sociodemographic characteristics including age, gender, and marital status, level of education, income, insurance status, and number of people sharing the house. Questions on awareness of patients' disease status, awareness of the need to take regular injections/medicine for prevention of ARF, and questions to assess patients' understanding of the importance of secondary prophylaxis were asked. For patients younger than 18 years, socioeconomic information and knowledge of guardians/parents were collected. Clinical data including type and number of valves affected, duration from RHD diagnosis, and history of previous valvular surgery were obtained from patients' hospital files.

Information about current or previous prophylaxis use was obtained from patients and/or their guardians and complemented by documentation from patients' clinical files. Patients were categorized as being “regular on prophylaxis” if they continuously received their monthly BPG injections from the time they were prescribed up until the time of recruitment. Patients who started on monthly injections and stopped taking the injections for more than 2 months consecutively (at the time of data collection) were termed as “stopped prophylaxis,” and those who had never been on any monthly injections were termed as “never started.”

### Isolation, Characterization, and Drug Sensitivity Testing for GAS

A trained research assistant collected throat swabs from all patients. Using a sterile swab, the posterior nasopharynx and the tonsillar arches were swabbed with special attention not to touch the cheeks, tongue, lips, or other areas of the mouth. Each collected swab was immediately immersed into a test tube containing Amies transport medium (Oxoid, England) (22). The samples were transported within 2 h to the Muhimbili National Hospital Central Pathology Laboratory for further processing.

At the laboratory, a dedicated laboratory research assistant processed all throat swab samples. Throat swabs were inoculated onto 5% sheep's blood agar plates, and the plates were incubated for 24 to 48 h at 37°C in aerobic environment. GAS isolates were identified based on the standard microbiological techniques, which include β-hemolytic activity on sheep's blood agar, small colony characteristics, Gram-positive cocci, catalase production–negative, and 0.04-U bacitracin disc susceptibility (22).

Antimicrobial susceptibility testing was done by using the Kirby–Bauer disc diffusion method according to the criteria set by Clinical Laboratory and Standard Institute (CLSI) version 2015 (23). Muller–Hinton agar supplemented with 5% sheep blood was used (23). Bacterial suspensions at a concentration of 10^5^ CFU/mL was inoculated on sheep blood Mueller–Hinton agar plates and incubated in aerobic environment for 24 h at 37°C. The antimicrobial discs of interest were chosen according to the prescribing patterns in local settings. The following discs with respective concentration were used: penicillin G (10 units), oxacillin (30 μg), ceftriaxone (30 μg), vancomycin (30 μg), erythromycin (15 μg), tetracycline (30 μg), ciprofloxacin (30 μg), chloramphenicol (30 μg), clindamycin (2 μg), and trimethoprim–sulfamethoxazole (25 μg). Zone-of-inhibition diameters were interpreted as sensitive, intermediate, and resistant according to the principles established by CLSI (23).

### Data Entry, Quality Assurance, and Analysis

Data were entered and analyzed using Statistical Package for Social Sciences computer software version 22 software (USA). For univariate analysis of quantitative variables such as age measures of central tendency including mean, mode, and median and measure of dispersion such as range, variance and standard deviation were used. For categorical data such as sex, level of education, and employment status, proportions were used. Non-parametric χ^2^ test was used to test statistical significance for frequency distribution of categorical data such as level of education vs. outcome of interest such as throat culture positivity. Student *t*-test was used to compare continuous variables among patients with and without positive throat swabs for GAS. Multiple logistic regression analysis was employed to determine the independent predictors of throat culture positivity. The results were of statistical significance when *P* < 0.05.

### Ethical Issues

Ethical approval to conduct the study was obtained from the Directorate of Research and Publications of the Muhimbili University of Health and Allied Sciences. All study participants had to sign an informed consent form before data were collected. For minors, consent was obtained from their parents or guardians, and assent was obtained from the patients.

## Results

### Sociodemographic Characteristics of the Study Participants

The mean ±*SD* age of study participants was 28.4 ± 16.5 years; it ranged from 5 to 75 years with median age of 24 years. More than half (53.6%) of the participants were younger than 25 years, and females made 58.2% of the study population; 59.3% of the participants had only attained primary education as their highest level of education; approximately a third were living in a family with seven or more people, 58.8% had no health insurance cover, and majority of the patients were unemployed (75.7%). Table 1 summarizes the sociodemographic characteristics of the study participants.

**Table 1:** Sociodemographic characteristics of the study participants (*N* = 194).

**Characteristic**	**Frequency**	**Percentage**
	**(*n*)**	**(%)**
**Age groups (years)**		
≤25	104	53.6
26–45	55	28.4
>45	35	18.0
**Sex**		
Males	81	41.8
Females	113	58.2
**Marital status**		
Single	65	33.5
Married	111	57.2
Divorced	7	3.6
Widowed	11	5.7
**Level of education**		
No formal education	10	5.2
Primary	115	59.3
Secondary	48	24.7
University	21	10.8
**Family size (Number)**		
≤6 people	127	65.5
≥7 people	67	34.5
**Mode of payment**		
Insurance	80	41.2
Out-of-pocket	114	58.8
**Average family income (TZS)**		
<70,000	113	58.2
70,000–310,000	52	26.8
>310,000	29	15.0
**Employment status**		
Employed	30	15.5
Unemployed	147	75.7
Retired	7	3.6
Student	10	5.2

*TZS, Tanzanian shillings*.

### Clinical and Other Characteristics of the Study Participants

Most of the study participants (84.5%) were aware of their medical condition, and they knew what they were suffering from. Slightly more than half (51%) of study participants knew about the need to take regular injections/medicine as prophylaxis, but only 17.5% knew the importance of the prophylaxis. Fifty-four (42.5%) participants were diagnosed within 1 year, whereas 32 (25.2%) were diagnosed between 1 and 3 years, and the rest of the study participants, 41 (32.3%), had been diagnosed more than 3 years prior to the study. Furthermore, 98 (50.5%) participants had single-valve diseases, whereas the remaining 96 (59.5%) had multiple valvular lesions (Table 2). Surgical intervention was done in 37 (19.1%) patients.

**Table 2:** Rheumatic valvular lesions in the total population.

**Valvular lesion**	**Frequency**	**Percent of total**
	**(*n*)**	**(%)**
Lone mitral regurgitation	52	26.8
Lone mitral stenosis	21	10.8
Mixed mitral regurgitation and stenosis	12	6.2
Lone aortic regurgitation	8	4.1
Lone aortic stenosis	4	2.1
Mixed aortic regurgitation and stenosis	1	0.5
**Other mixed valvular patterns**		
Mitral and aortic	39	20.1
Mitral and tricuspid	22	11.3
Mitral and aortic and tricuspid	35	18.0

### ARF Prophylaxis Status

Among 194 patients interviewed, 58 (29.9%) were on regular prophylaxis at the time of data collection, 39 (20.1%) had stopped prophylaxis, and 97 (50.0%) had never been on prophylaxis since diagnosis. Of the 58 patients who were on regular prophylaxis, 33 (56.9%) were on 4-weekly regimen, and the remaining 25 (43.1%) were on 3-weekly regimen.

### Throat Colonization and Antimicrobial Susceptibility of GAS Isolated From Study Participants

**Figure 1:**
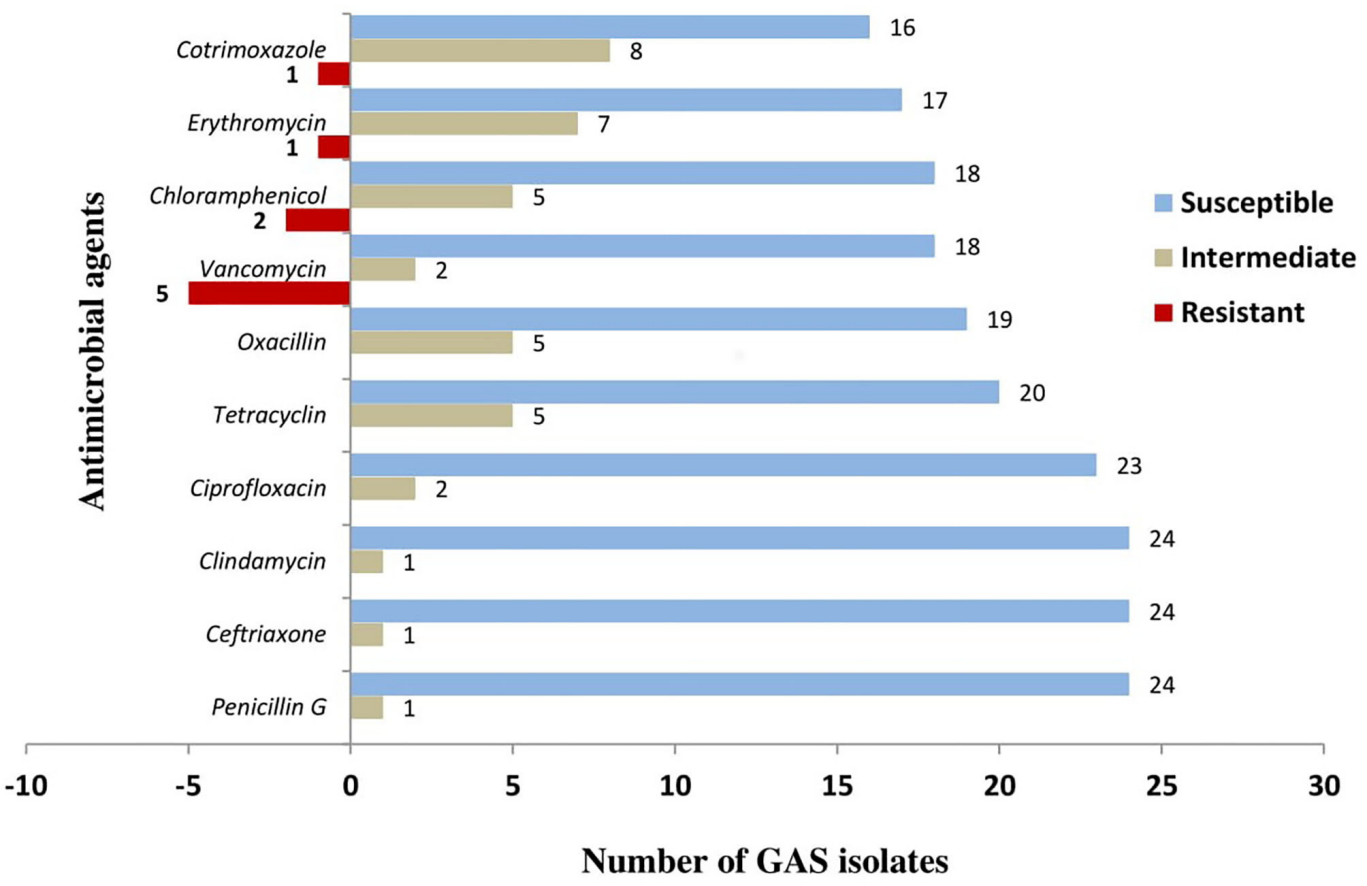
Antimicrobial susceptibility of GAS isolated from RHD patients.

In the total study population, throat culture results of 25 patients were positive for GAS, giving a GAS throat colonization rate of 12.9%. GAS isolated from the 25 patients were mostly susceptible to BPG (24/25, i.e., 96% susceptible), ceftriaxone (24/25, i.e., 96% susceptible), and clindamycin (24/25, i.e., 96% susceptible). GAS isolates were found to show highest resistance toward vancomycin (5/25, i.e., 20% resistance) and chloramphenicol (2/25, i.e., 8% resistance). There were also high intermediate susceptibilities toward most commonly used antimicrobial agents including oxacillin (20%), erythromycin (28%), and cotrimoxazole (32%). Figure 1 shows the susceptibility patterns of GAS toward the eight antibiotics that were tested. The negative numbers shown in Figure 1 represent the number of isolates that were resistant to the respective antibiotics.

### Factors Associated With GAS Throat Colonization Among Study Participants

Patients with positive throat culture did not differ from those with negative culture with regard to age distribution, gender, level of education, number of people in the household, and family income, all *p* > 0.05 (Table 3). They also did not differ in terms of knowledge and understanding of importance of ARF prophylaxis, *p* > 0.05 for both (Table 3). Although not statistically significant, patients with positive culture had higher proportions of uninsured (68 vs. 57.4%), unemployed (38 vs. 22.5%), those unaware of their medical condition (24 vs. 14.2%), and patients with multiple valve disease (60 vs. 47.9%) (Table 3).

**Table 3:** Sociodemographic, clinical, and other characteristics in relation to GAS throat colonization.

**Characteristic**	**Culture**	**Culture**	***p*-value**
	**negative**	**positive**	
	**(*n* = 169)**	**(*n* = 25)**	
Age <25 (years)	89 (52.7)	13 (52)	0.559
Female gender	97 (57.4)	16 (64)	0.345
Primary or less level of education	110 (65.1)	15 (60)	0.387
≥7 people in the household	59 (34.9)	8 (32)	0.483
Not insured	97 (57.4)	17 (68)	0.217
Family income TZS <70,000	97 (57.4)	16 (64)	0.345
Unemployed	38 (22.5)	8 (38)	0.211
Didn't know about RHD suffering	24 (14.2)	6 (24)	0.165
Didn't know about ARF prophylaxis	84 (49.7)	11 (44)	0.376
Didn't know the importance of prophylaxis	139 (82.2)	21 (84)	0.545
Had surgical intervention	30 (17.7)	6 (24)	0.331
Had multiple valve disease	81 (47.9)	15 (60)	0.181
**Prophylaxis status**			
On regular prophylaxis	52 (89.7)	6 (10.3)	0.029
Stopped prophylaxis	29 (74.3)	10 (26)	
Never been on prophylaxis	88 (90.7)	9 (9.3)	

*TZS, Tanzanian shillings; RHD, rheumatic heart disease; ARF, acute rheumatic fever*.

With regard to prophylaxis status, patients who had stopped prophylaxis had significantly higher proportion with positive cultures (26%) when compared to those who were on regular (10.3%) and those who had never started (9.3%), *p* = 0.029 (Table 3).

Prophylaxis status and other factors that were weakly associated (*p* < 0.5) with culture positivity were entered into a logistic regression model to determine the factors that are independently associated with GAS culture-positive results. After removing interacting variables, the following were entered in the final model: gender, insurance status, number of people in the household, disease awareness, prophylaxis status, multiple valvular disease, and history of previous surgery. Having stopped prophylaxis was the only factor that was independently associated with positive culture results in multivariate logistic regression analysis, odds ratio (OR) = 3.26 [95% confidence interval (CI) = 1.04–10.24], *p* = 0.043 (Table 4). Specifically, compared to patients on regular ARF prophylaxis, patients who stopped prophylaxis were 3.26 times more likely to have positive throat culture results independent of gender, disease awareness, insurance status, number of diseased valves, or previous valvular surgery (Table 4).

**Table 4:** Multivariate logistic regression analysis of factors associated with GAS colonization among RHD patients.

**Variable**	**Univariate analysis**		**Multivariate analysis**	
	**OR (95% CI)**	***p-*value**	**OR (95% CI)**	***p-*value**
Female sex	1.32 (0.55–3.16)	0.533	1.31 (0.53–3.31)	0.547
Uninsured	1.58 (0.65–3.86)	0.318	1.62 (0.61–4.03)	0.348
Living >7 people in the household	0.88 (0.36–2.15)	0.775	0.73 (0.28–1.93)	0.524
Unaware of disease condition	1.91 (0.69–5.26)	0.212	2.42 (0.77–7.59)	0.129
**Prophylaxis status**				
Regular on prophylaxis	Reference		Reference	
Stopped prophylaxis	2.99 (0.99–9.06)	0.053	3.26 (1.04–10.24)	0.043
Never started	0.89 (0.30–2.63)	0.828	0.98 (0.32–3.02)	0.975
Multivalve disease	1.63 (0.69–3.83)	0.263	1.87 (0.74–4.67)	0.189
History of previous surgery	1.41 (0.52–3.81)	0.503	1.87 (0.62–5.59)	0.265

## Discussion

GAS throat colonization is a well-known risk factor for development of sore throat and subsequent ARF in the general population (24), but more importantly among people with previous history of ARF and in those with RHD (3). This is more serious for those who are particularly at a greater risk due to a number of factors including their genetic susceptibility, which renders them more sensitive to infection with a rheumatogenic strain of GAS (25). Only few studies from sub-Saharan Africa have reported the prevalence of GAS colonization in the general population (8–10, 22, 26), in patients with pharyngotonsillitis (27, 28), and among RHD patients (7). The present study therefore adds to the current knowledge on RHD in the region by demonstrating that among RHD patients attending care at a tertiary health facility in Tanzania, throat GAS colonization is present in 12.9% and is independently associated with stopping ARF prophylaxis among these patients.

The 12.9% prevalence of throat GAS colonization found in this study is much higher than that found among 233 children with chronic RHD attending care at a cardiac clinic in Addis Ababa, Ethiopia (7), but within the reported prevalence among school-going children in sub-Saharan Africa (8–10). Of note, the prevalence of GAS positivity in the study by Zegeye et al. (7) was 6.9%, and the difference between the two studies can mainly be attributed to the fact that all patients in the Ethiopian study were on ARF prophylaxis as compared to the present study where prophylaxis was taken regularly by 29.9% of the total studied population. The deleterious effects of GAS colonization among patients with RHD have been well-documented (29). The finding of our study is therefore clinically very relevant, meaning that ~13% of RHD patients in our setting are at increased risk of worsening of their disease and therefore progression toward heart failure and other complications brought about by RHD (30).

The finding that stopping ARF prophylaxis is independently associated with positive GAS throat culture results is in agreement with previous reports in literature (7, 27, 29, 31). In the study by Zegeye et al. (7) from Ethiopia, children who missed at least one prophylaxis within the last 6 months had a higher culture positivity rate than those who did not miss any scheduled prophylaxis. In the present study, having stopped prophylaxis increased the likelihood of GAS colonization up to three times. The explanation for increased GAS colonization among patients who stop prophylaxis is to a larger extent clear, because interrupting the dose or stopping the prophylaxis means the patient will not have the required level of the antibiotic in blood that is necessary to prevent GAS throat colonization (26).

The finding that less than a third (29.9%) of patients with RHD in our setting was on regular prophylaxis is alarming. Furthermore, the fact that half of the study participants (50%) had never been on any ARF prophylaxis raises even more concerns. Although reasons for not being on regular prophylaxis were not systematically studied in the present study, it is unlikely that the patients who were never on prophylaxis had clinically relevant reasons not to be on prophylaxis against ARF. Ideally, any patient with confirmed RHD needs to be on prophylaxis at least for some period of time as per guidelines (32). This is also true for those who stopped taking their prophylaxis. The reasons for stopping prophylaxis are most likely multifactorial, and further research focusing on physicians-related factors, patients-related factors, and factors related to our health care delivery system need to be studied. All these factors have been found to influence prescribing practice of prophylaxis and adherence among RHD patients elsewhere (6, 33–35).

Using *in vitro* susceptibility assay, GAS isolated from RHD patients in this study were almost 100% susceptible to penicillin G. This finding is similar to many previous studies in documented literature, and it is at least encouraging to know that despite being in the market for more than eight decades, penicillin is still performing well in terms of GAS susceptibility (29–31). This may be due to penicillin being limited to use only in few number of diseases including pharyngitis, syphilis, ARF prophylaxis, and so on. There has been, however, reports of GAS resistance to penicillin (32, 36), and more care should be taken to avoid risk factors that may lead to increased chances to develop resistance to penicillin in our setting. Factors such as unreliable and interrupted doses, as well as poor quality of penicillin, have been reported to increase GAS resistance to the drug *in vitro* as well as *in vivo* studies (37). The pattern of reduced GAS susceptibility (intermediate results) and resistance toward vancomycin, chloramphenicol, erythromycin, and cotrimoxazole observed in the current study is similar to other studies, most likely caused by factors such as increased frequency and irrational use of these antibiotics (38).

We found in this study that knowledge on the need and the importance of being on prophylaxis is low among patients with RHD. This has negative implications as far as the management of RHD is concerned, considering the chronic nature of the disease and the need for patients to take regular medications and to follow regular visits to the health facilities. The low knowledge could have been one of the contributing factors to stopping the prophylaxis (although this was not actively assessed in this population), as well as could have affected the adherence status in this population. In a brief communication by Bergmark et al. (5) reporting the burden of disease and barriers to the diagnosis and treatment of GAS pharyngitis in Dar es Salaam, the clinicians who were interviewed stated that identifying and treating streptococcal pharyngitis were not their priority. This further explains the multifactorial nature of the factors related to overall poor management of RHD patients in our local setting. This calls for more efforts to increase awareness of RHD management and the importance of clinicians to follow guidelines.

The baseline sociodemographic characteristics seen in this study population are similar to that found in many other previous studies mostly consisting of young, predominantly female patients with high unemployment rates (18, 39, 40). Of note, the mean age of the present study population was 28.4 years, and women comprised 58.2% of the study population. Furthermore, the proportion of patients with primary or less education was 64.5%, and more than three quarters of the patients were unemployed. This picture represents the well-known population at risk of GAS pharyngitis, ARF, and RHD (18, 39, 40). Contrary to previous findings (7), none of the socioeconomic factors studied in this population was associated with throat GAS colonization. The differences in the findings between the present and previous studies could be due to differences in methods used to assess risks, but also, it is possible that the present study was not adequately powered to detect these associations, and only trends were seen toward more patients with poor socioeconomic indices to be aggregated in the group of patients with positive throat culture results (Table 2).

## Conclusion

The prevalence of GAS throat colonization is high among this population and is associated with stopping prophylaxis. The proportion of patients on regular secondary prophylaxis is unacceptably low, and interventions should target both patients' and physicians' barriers to effective secondary prophylaxis.

## Limitations

Because data collection was done from a single referral center, the findings from this study cannot be generalized to other centers, especially in remote areas of Tanzania where access to health care is limited. It is therefore possible that an even worse picture of secondary prophylaxis would be found in these remote areas. We did not actively study the presence of active sore throat and ARF in this study population; however, all patients were outpatients coming for their regular clinic follow-up and therefore largely asymptomatic. We also did not perform polymerase chain reaction typing to validate the findings of the Lancefield classification due to limited budget of the study. As it is the case for all cross-sectional studies, it is impossible to make further inferences that non-adherence to prophylaxis is a predictor of GAS throat colonization; thus, only associations can be concluded from this study. The limited sample size may have contributed to lack of associations between some variables and GAS throat colonization because the sample size calculation was based on prevalence only.

## Data Availability Statement

The datasets generated for this study are available on request to the corresponding author.

## Ethics Statement

The studies involving human participants were reviewed and approved by Muhimbili University of Health and Allied Sciences Research and Publication Committee. The patients/participants provided their written informed consent to participate in this study.

## Author Contributions

SW, PC, and AK conceived, designed the study, and participated in data analysis. SW, DK, and MJ conducted data collection. All authors participated in developing the manuscript and approved the final version.

## Conflict of Interest

The authors declare that the research was conducted in the absence of any commercial or financial relationships that could be construed as a potential conflict of interest.

## Abbreviations

ARF: Acute Rheumatic Fever
BPG: Benzathine Penicillin G
CLSI: Clinical Laboratory and Standard Institute
GAS: Group A β-hemolytic Streptococcus
RHD: Rheumatic Heart Disease
WHO: World Health Organization.
