# Supplementary material for: Throat Colonization and Antibiotic Susceptibility of Group a β-Hemolytic Streptococci Among Rheumatic Heart Disease Patients Attending a Cardiac Referral Hospital in Tanzania, a Descriptive Cross-Sectional Study
Source: Front Surg. 2020 Sep 17;7:57. doi: 10.3389/fsurg.2020.00057 (PMC7527597; doi:10.3389/fsurg.2020.00057)
Supplement: Supplementary file 1 [file Table_1.DOCX]

# APPENDIX II: CASE REPORT FORM (CRF)

**THROAT CULTURE COLONIZATION AND ANTIBIOTIC SUSCEPTIBILITY OF GROUP A β-HEMOLYTIC STREPTOCOCCI IN RHEUMATIC HEART DISEASE PATIENTS AT JAKAYA KIKWETE CARDIAC INSTITUTE.**

## **Questionnaire for patients/parents/guardians**

Patient Code………………..Date: ..............................

**Part A**: **Socio-demographic information**

1. Patient age (years)..............

2. Patient gender:

Male [ ]

Female [ ]

***For patients <18 years the following questions to be answered by guardians/parents**

3. **Marital status:**

Single [ ] Married [ ]

Divorced [ ] Widowed [ ]

4. Residence:

Within Dar es Salaam [ ] specify street ………….

Outside Dar es Salaam [ ] Specify region………………

5. Level of education:

Primary education [ ] Secondary education [ ]

University education [ ] Tertiary [ ]

No formal education [ ]

6. Employment status:

Employed [ ] unemployed [ ]

Self-employed [ ] retired [ ]

Student others [ ] (specify). ................

7. Family average monthly income:

< TZS 70 thousand [ ] TZS 70-310 thousands [ ]

>TZS 310 thousands [ ] Declined to answer [ ]

8. Mode of payment:

Insurance [ ] Out of pocket [ ]

Exemption [ ]

9. Number of people living in the household:

<6 [ ]

>7 [ ]

10. Family history of RHD

Yes [ ]

No [ ]

**Part B: Awareness of prophylaxis against GAS**

11. Do you know what you/your child are suffering from? Yes [ ] No [ ]

If yes briefly state what it is……………………………………………………….

13. Do you know that you/your child are supposed to be getting monthly injections?

Yes [ ] No [ ]

13. Are you receiving monthly or three weekly injections for secondary prophylaxis of RHD?

Yes [ ] No [ ]*

14. Do you know the name of the injection which you/your child is getting

Yes [ ] No [ ]

If Yes mention the drug……………………………………………..

15. Do you know the importance of these monthly injections?

Yes [ ] No [ ]

If Yes briefly explain ………………………………………………………………

**If no check on the Patient records to see if they are on oral prophylaxis*

**Part C: Adherence (Answer yes= 1 or No = 0).**

15. Do you sometimes forget to get monthly injections? Yes [ ] No [ ] NA [ ]

16. People sometimes miss taking their medications for reasons other than forgetting. Thinking over the past six months, were there any months when you/your child did not get monthly injections? Yes [ ] No [ ]

If Yes How many doses did you/your child miss................

Mention the reason(s) for missing monthly injection

- 1. ………………………………………………………….
  2. ………………………………………………………….
  3. ………………………………………………………….
  4. ………………………………………………………….

17. Have you/your child ever cut back or stopped getting monthly injections without telling your doctor because you/your child felt worse when you got the injections? Yes [ ] No [ ]

18. Did you/your child get injection last month? Yes [ ] No [ ] NA [ ]

19. When you travel do you sometimes forget to get the monthly injection? Yes [ ] No [ ]

20. When you feel like you/your child’s symptoms are under control do you sometimes stop getting monthly injections? Yes [ ] No [ ]

21. Getting injections every month is a real inconvenience for some people. Do you/your child ever feel hassled about sticking to your treatment plan? Yes [ ] No [ ]

22. How often do you have difficulty remembering get you/your child monthly injection? (A=1, BCDE=0)

___A. Never/rarely

___B. Once in a while

___C. Sometimes

___D. Usually

___E. All the time

## Part D: Information from patient files

23. Diagnosis.....................................................

24. Type of Lesion...........................................

25. Surgical intervention done Yes [ ] No [ ]

26. If Yes, type of Surgical intervention………………………………….

## Part E: Laboratory Results

27. Culture positivity i) Positive [ ] ii) Negative [ ]

**Template for filling in GAS susceptibility results to the tested antibiotics**

| Name of Antibiotic | Zone of Inhibition Diameter (mm) | Interpretation of Results. (Interpreted as Resistant, Intermediate or Susceptible) |
| --- | --- | --- |
| Penicillin G (10 units) |  |  |
| Oxacillin (30μg) |  |  |
| Ceftriaxone(30μg) |  |  |
| Vancomycin (30μg) |  |  |
| Erythromycin (15μg) |  |  |
| Tetracycline (30μg) |  |  |
| Ofloxacin (5μg) |  |  |
| Chloramphenicol (30μg) |  |  |
| Clindamycin (2μg) |  |  |
| Trimethoprim- sulfamethoxazole (1.25/23.75μg) |  |  |
